# Method to Minimize the Errors of AI: Quantifying and Exploiting Uncertainty of Deep Learning in Brain Tumor Segmentation

**DOI:** 10.3390/s22062406

**Published:** 2022-03-21

**Authors:** Joohyun Lee, Dongmyung Shin, Se-Hong Oh, Haejin Kim

**Affiliations:** 1 Department of Electrical and Computer Engineering, Seoul National University, Seoul 08826, Korea; lleezz@snu.ac.kr (J.L.); shinsae11@snu.ac.kr (D.S.); 2 Division of Biomedical Engineering, Hankuk University of Foreign Studies, Yongin 17035, Korea; 3 College of Science & Technology, Hongik University, Sejong 30016, Korea

**Keywords:** brain tumor, semantic segmentation, uncertainty quantification, attention mechanism

## Abstract

Despite the unprecedented success of deep learning in various fields, it has been recognized that clinical diagnosis requires extra caution when applying recent deep learning techniques because false prediction can result in severe consequences. In this study, we proposed a reliable deep learning framework that could minimize incorrect segmentation by quantifying and exploiting uncertainty measures. The proposed framework demonstrated the effectiveness of a public dataset: Multimodal Brain Tumor Segmentation Challenge 2018. By using this framework, segmentation performances, particularly for small lesions, were improved. Since the segmentation of small lesions is difficult but also clinically significant, this framework could be effectively applied to the medical imaging field.

## 1. Introduction

Gliomas are the most common primary brain tumors and are classified by grading. Glioblastoma is the most aggressive glioma associated with short-term survival compared to low-grade glioma. Gadolinium (Gd) enhancement MR imaging is the first choice in diagnostic modality for distinguishing heterogeneous tissue. Modality can effectively represent the Gd-enhancing tumor, non-enhancing tumor, and necrotic and peritumoral edematous areas. The proper segmentation of these heterogeneous areas is crucial for surgery and radiotherapy [1,2,3]. However, manual segmentation in the clinical field is a tedious and time-consuming task that expert neuroradiologists can only accomplish. Moreover, intraobserver and interobserver variabilities have been reported to be over 20% for the manual segmentation of brain tumors [2,4].

To overcome the aforementioned limitations, numerous automated glioma segmentation methods have been adapted to diagnose brain tumors more accurately, rapidly, and consistently [5,6,7,8,9]. Since 2012, the worldwide Multimodal Brain Tumor Image Segmentation (BraTS) Challenge was established to facilitate the progress of automated glioma segmentation [1,2,4]. BraTS Challenge 2018 provides a large dataset consisting of multimodal magnetic resonance imaging (MRI) scans of patients with low-grade and high-grade glioma and manually segmented results. A single segmentation was conducted by multiple raters and experienced neuro-radiologists to minimize inter-rater variability. Many deep learning methods have been developed and published to segment gliomas based on this BraTS dataset [10,11,12,13]. These deep learning methods commonly focus on diagnostic accuracy as well as speed (inference time) and cost (computational complexity). Most top-ranking models in the BraTS18 Challenge were ensembles of 3-dimensional (3D) convolutional neural networks (CNN), which achieved excellent performance and resulted in huge computational cost and time. [11,12,13]. Myroneko [11] designed an ensemble model comprising ten 3D CNNs and achieved first place in the BraTS18 Challenge. On the contrary, several studies have focused on effectiveness instead of accuracy. Chen et al. [10] designed an effective 3D CNN that significantly reduced cost and time while achieving comparable segmentation accuracy.

However, the AI diagnosis method often generates incorrect predictions. Therefore, AI has not been widely applied in the clinical field yet [14,15]. There have been many studies that seek to overcome this dilemma [16,17,18,19,20]. To fully trust the prediction of AI, other metrics may be needed in addition to accuracy only [14,21]. Several deep learning studies recently suggested an uncertainty quantification method for accurate lesion detection, which can be a significant indicator to convince prediction [14,15,20]. Uncertainty in this context means that the prediction result by AI is uncertain. Therefore, calculating uncertainty measures in lesion segmentation is essential for correcting prediction errors. Kwon et al. [20] designed a neural network to quantify prediction uncertainty with two different moment-based metrics (aleatoric and epistemic). This approach showed that the uncertainty quantification method provides additional insights for accurate diagnosis in ischemic stroke lesion segmentation. Nair et al. [14] designed a 3D Multiple Sclerosis segmentation CNN that quantifies and exploits four different uncertainty measurements, including mutual information and entropy. Uncertain predictions were eliminated based on pixel-wise uncertainty measures. Therefore, uncertainty filtering improves the true positive rate and reduces the false detection rate on remaining predictions. However, each uncertainty measure should have a threshold. This threshold is a specific value to decide whether the corresponding prediction should be excluded. Finding these values is a heuristic task and requires numerous experiments [14].

In this study, a new CNN framework was designed for brain tumor segmentation to exploit various uncertainty measures effectively. We applied the previously published baseline CNN into our framework and demonstrated performance improvements by exploiting the uncertainty measures. This study aims (1) to find out uncertain predictions by quantifying four different uncertainty measures and (2) to correct the uncertain predictions by exploiting integrated measures.

## 2. Materials and Methods

### 2.1. Dataset

**Figure 1:**
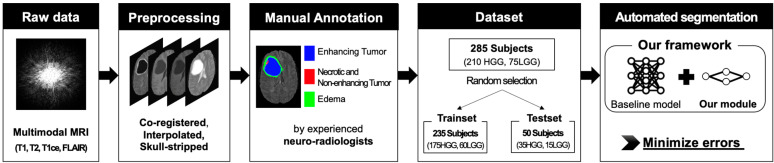
Flow diagram of our study. Brain tumor segmentation on MRI was automatically performed, and three types of lesions (enhancing tumor, necrotic/non-enhancing tumor, and edema) were distinguished. The proposed framework can minimize the error of the baseline model. To evaluate the framework, we used a public dataset, BraTS18. T1 = T1-weighted; T1ce = T1 contrast-enhanced; T2 = T2-weighted; FLAIR = Fluid Attenuated Inversion Recovery; HGG = high-grade glioma (glioblastoma); LGG = low-grade glioma.

A brief explanation of our study is summarized in Figure 1. We used a public dataset, BraTS18, which is provided in the Multimodal Brain Tumor Segmentation Challenge [1,2,4]. The dataset comprises 3T multimodal MRI scans and manual lesion annotations (ground truth) by expert neuroradiologists following the same annotation protocol. The total number of patients were 285, and patients’ final diagnoses were glioblastoma (HGG, *n* = 210) and low-grade glioma (LGG, *n* = 75). Multimodal MRI of the patients comprised four MRI contrasts, and T1-weighted (T1), contrast-enhanced T1-weighted (T1ce), T2-weighted (T2), and Fluid Attenuated Inversion Recovery (FLAIR) images were acquired with various scanners from multiple institutions (*n* = 19). The MRI images have been preprocessed (co-registered to the same anatomical template, interpolated to the same resolution (1 mm^3^), and skull-stripped), and each contrast image covers the entire brain (240 × 240 × 155 mm^3^). All images have been manually segmented by one to four raters, and skilled neuroradiologists confirmed the annotations. Each image has manually annotated labels indicating a Gd enhancing tumor, necrotic/non-enhancing tumor core, and peritumoral edema.

Automated segmentation algorithms for the BraTS18 challenge were used to find the segmentation map of the brain tumor’s subregions. The subregions are as follows: 1. the enhancing tumor (ET); 2. the tumor core (TC)l; and 3. the whole tumor (WT). ET has more hyperintense lesions than normal brain parenchyma in the T1ce and T1 images. The necrotic and non-enhancing tumors show lower intensity in T1ce when compared to T1. TC is an actual tumor bulk, including enhancing tumors, necrotic, and non-enhancing tumors. The peritumoral edemas are hyperintense lesions in FLAIR. WT includes the whole extent, including TC and peritumoral edemas.

We randomly divided the dataset to train and evaluate the networks. The training set included 235 patients (175HGG and 60LGG), and the testing set included 50 patients (35HGG and 15LGG) to assess the segmentation performance of the proposed model.

### 2.2. Baseline Model

The goal of our study is to upgrade the baseline model. The proposed framework is designed to find out mistakes of baseline prediction and to correct them. The baseline models in this study were well-known brain tumor segmentation models that focus on effectiveness [10]. These models achieved real-time segmentation by significantly decreasing computational cost and time.

### 2.3. Uncertainty Quantification

**Figure 2:**
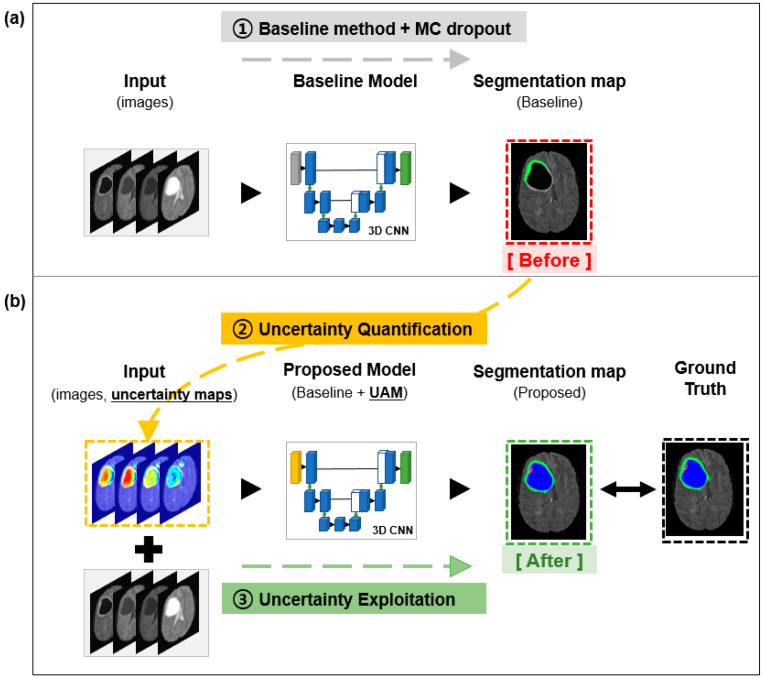
Overview of the proposed framework. (**a**) Feedforward multimodal MRI images into a baseline model with Monte Carlo dropout and generate a baseline segmentation map (Step. 1) (**b**) Quantify uncertainty values of baseline segmentation map by four different measures (Step. 2). Feedforward both images and uncertainty maps into the proposed model and generate proposed segmentation map (Step. 3); MC dropout = Monte Carlo dropout; 3D CNN = 3-dimensional convolutional neural network; UAM = uncertainty attention module.

The metrics of uncertainty were carefully selected based on adaptability and effectiveness. It should be adaptable to the deep learning algorithm and should be easily measurable. Based on these criteria, Aleatoric, Epistemic, Entropy, and Mutual Information are selected. To measure the uncertainty of the baseline prediction, a statistical technique, Monte Carlo (MC) dropout sampling, was integrated with the baseline model. This MC dropout sampling is a well-known statistical technique used to estimate the reliability of the prediction by quantifying the uncertainty of the prediction [22]. We adopted the aforementioned baseline models for our study [10] (Figure 2a). These models can only generate semantic segmentation maps, but we applied MC dropout, randomly disconnecting 10% of the neuronal connections, after every convolution layer. An input image was forwarded T (7) times at inference time while applying dropout, generating T segmentation samples. Because of dropout sampling, the T segmentation samples were slightly different from each other. These samples were used to generate the four different uncertainty maps (Aleatoric, Epistemic, Entropy, and Mutual Information) [14,22,23,24]. The final outputs of the baseline models were segmentation maps and uncertainty maps estimated by four different mathematical measures.

To generate pixel-wise UMs for image segmentation, we used the samples of the segmentation results generated from the baseline model. The fully trained baseline model with training dataset D was used to generate segmentation samples. Each input image (x) is 3-dimensional in size (240×240×155) and has about 9 millions (N) voxels. Since all voxels were calculated by using the equations below, the three-dimensional voxel values were simplified to one dimension. For the $i^{th}$ input image $x_{i}$ and the $t^{th}$ sampled network parameters with MC dropout $\theta_{t}$, the segmentation probability of class c for the $j^{th}$ voxel of the $i^{th}$ image $p(y_{it}^{j}=c|x_{i},\;\theta_{t})$ was generated. Without a loss of generality, we denote $x\;:=x_{i}$ and $y\;:=y_{i}^{j}$ for the following definitions.

#### 2.3.1. Aleatoric Uncertainty

Aleatoric uncertainty captures the inherent randomness in the observation, which can be expressed as follows [23,24].

(1) $$Aleatoric\left(y\;|\;D\right):=\;\underset{c}{\sum }E_{p\left(\theta \;|\;D\right)}\left[p\left(y=c\;|\;x,\theta \right)-p{\left(y=c\;|\;x,\theta \right)}^{2}\right]\;\approx \;\overset{T}{\underset{t}{\sum }}\underset{c}{\sum }p(y_{t}=c\;|x,\theta_{t})\left[1-p(y_{t}=c\;|x,\theta_{t}\right)]$$

#### 2.3.2. Epistemic Uncertainty

Epistemic uncertainty explains model uncertainty. This can be estimated by the following [23,24]:

(2) $$Epistemic\left(D\right):=\;\underset{c}{\sum }E_{p\left(\theta \;|\;D\right)}{\left[p\left(y=c\;|\;x,\theta \right)-q\left(y=c|x\right)\right]}^{2}\;\;\approx \;\overset{T}{\underset{t}{\sum }}\underset{c}{\sum }\left[p(y_{t}=c\;|\;x,\theta_{t}\right)-\bar{p}(y=c\;|x,\theta ){]}^{2}$$

where the following is obtained.

$$\bar{p}\left(y_{t}=c|x,\theta \right):=\frac{1}{T}\overset{T}{\underset{t=1}{\sum }}\hat{p}\left(y_{t}=c|x,\theta_{t}\right)$$

#### 2.3.3. Entropy

Entropy shows how much information is in the model’s predictive density function [14,22]. The entropy can be approximated by the MC samples as follows.

(3) $$Entropy\left(D\right):=-\underset{c}{\sum }p\left(y=c\;|\;x,D\right)\log\;p(y=c\;|\;x,D)\;\approx -\underset{c}{\sum }\bar{p}(y=c\;|\;x,\theta )\log\bar{p}(y=c\;|\;x,\theta )$$

#### 2.3.4. Mutual Information

The mutual information of two variables is the measurement of the mutual dependence between the two variables [14,22]. Mutual information can be approximated by the difference between the expectation of model entropies and the expected prediction entropy.

(4) $$\;Mutual\;Information\left(y;\theta \;|\;D\right):=H\left(y\;|\;D\right)-E_{p\left(\theta \;|\;D\right)}\left[H(y\;|\;x,\theta \right)]\;\approx -\underset{c}{\sum }\bar{p}(y=c\;|\;x,\theta )\log\bar{p}(y=c\;|\;x,\theta )\;+\frac{1}{T}\overset{T}{\underset{t=1}{\sum }}\underset{c}{\sum }p(y=c\;|\;x,\theta )\log p(y=c\;|\;x,\theta )$$

### 2.4. Uncertainty Exploitation

**Figure 3:**
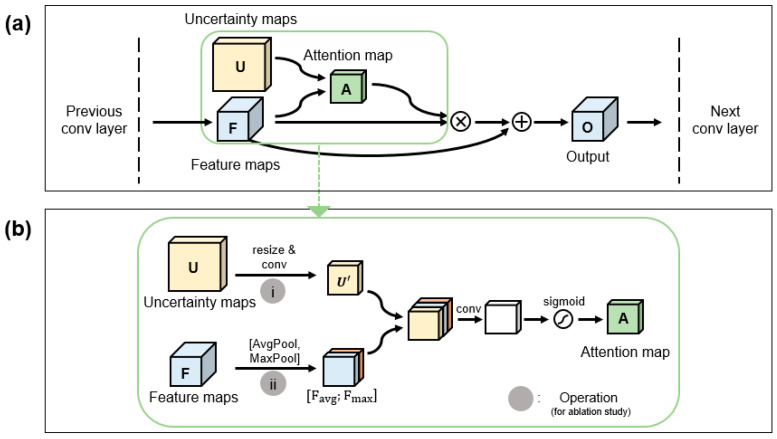
Architecture of uncertainty attention module (UAM). This subnetwork module can effectively exploit uncertainty maps by applying an attention mechanism. This module is plugged into any neural network. This architecture, including the operations, was highly optimized by an ablation study. Conv = convolution; AvgPool = average pooling; MaxPool = max pooling; F_avg_ = feature maps from average pooling operation; F_max_ = feature maps from max pooling operation. (**a**) UAM architecture overview. (**b**) Specific architecture of green box.

By following our framework, we had four different UMs describing these predictions as uncertain and highly likely to be errors. We considered exploiting these meaningful maps further to design a more accurate model. We brought this idea to the training model by thinking of human beings who learn from their mistakes. The Ums, which are the weaknesses of the model, were exploited to train the deep learning model. The uncertainty exploitation method is illustrated in Figure 2b, and detailed implementation is described in Figure 3.

Our work directly utilized UMs by using a newly designed module called the uncertainty attention module (UAM). The proposed model is based on the baseline model in which UAM was plugged in. The overall structure of UAM is described in Figure 3a, and specific operations are summarized in Figure 3b. As the first step, an uncertainty block, *U* ($\in R^{4\times H^{*}\times W^{*}}$ where 4, $H^{*},\;\;\mathrm{and}\;W^{*}$ refer to the four different UMs, the height of the input image, and the width of the input image, respectively), was generated by concatenating the four UMs along the channel’s axis. The feature map, *F* ($\in R^{C\times H\times W}$ where *C*, *H*, and *W* refer to the channel, height, and width of the intermediate feature map, respectively), from the previous convolution block was combined with the uncertainty block to generate an attention map, $A\;\in R^{1\times H\times W}$. Since the uncertainty block and the feature map differed in size, the uncertainty block was resized and denoted as $U_{resized}$. $U_{resized}$ was forwarded into a 3 × 3 convolutional layer and denoted as $U^{\prime }$. Simultaneously, the feature map from the previous layer (F) was forwarded into the average pool layer and maxpool layer, which are denoted as $F_{avg}$ and $F_{max}$. All maps ($U^{\prime },\;F_{avg},\;F_{max}$) were concatenated along the channel axis. The concatenated map was convolved by a 3×3 convolutional layer and normalized by a unipolar sigmoid function to generate the attention map. This can be summarized as follows:

(5) $$A\left(F,U\right)=\sigma \left(f^{3\times 3}\left[f^{3\times 3}\left(U_{resized}\right);AvgPool\left(F\right);MaxPool\left(F\right)\right]\right)\;=\sigma \left(f^{3\times 3}\left[U^{\prime };\;F_{avg};\;F_{max}\right]\right)$$

where σ denotes the unipolar sigmoid function, $f^{3\times 3}$ denotes the 3×3 convolution layer, and [ ] denotes the concatenation operation. 

The final output *O*$\;\in R^{C\times H\times W}$, which is a refined feature map, was computed with a skip connection to reduce gradient vanishing [25,26]:

(6) O(F,U)=(F⊗A)⊕F

where ⊕ denotes element-wise summation, and ⊗ denotes element-wise multiplication. In this work, UAM was plugged in the baseline model in every unit (i.e., every five layers).

### 2.5. Model Training

In this experiment, there were various hyperparameters, including loss function, optimizer, and data augmentation methods for the framework. All hyperparameters used in this experiment are described below. 

The loss function for the model was Dice-Coefficient Loss [27] and is defined by the following:

(7) $$(Q2-1)\;Dice\;Coefficient\;Loss=1-\frac{2\sum_{j}^{N}p_{j}g_{j}}{\sum_{j}^{N}p_{j}^{2}+\sum_{j}^{N}g_{j}^{2}}$$

where $p_{j}$ is the binary value of the $j^{th}$ voxel in the segmentation output, (Q2−1) $g_{j}$ is the binary value of the $j^{th}$ voxel in the ground truth, and N is the number of voxels. The following hyperparameters were applied for the training model: Adam optimizer (coefficients [0.5, 0.999]), $L_{2}$ norm (weight decay rate: 0.00001), batch size (8), epochs (200), initial learning rate (0.001), number of class c(4), and number of MC sampling T (7). Overfitting was mitigated by (i) random cropping from 240 × 240 × 155 voxels to 96 × 96 × 96 voxels; (ii) random flipping across coronal, sagittal, and axial planes (probability: 0.5); and (iii) random rotations between (Q2−2) [$-10^{{}^{\circ}},\;10^{{}^{\circ}}$], (iv) random intensity shifts between [−0.1, 0.1], and (v) random scaling between [0.9, 1.1]. 

## 3. Results

### 3.1. Uncertainty Quantification

**Figure 4:**
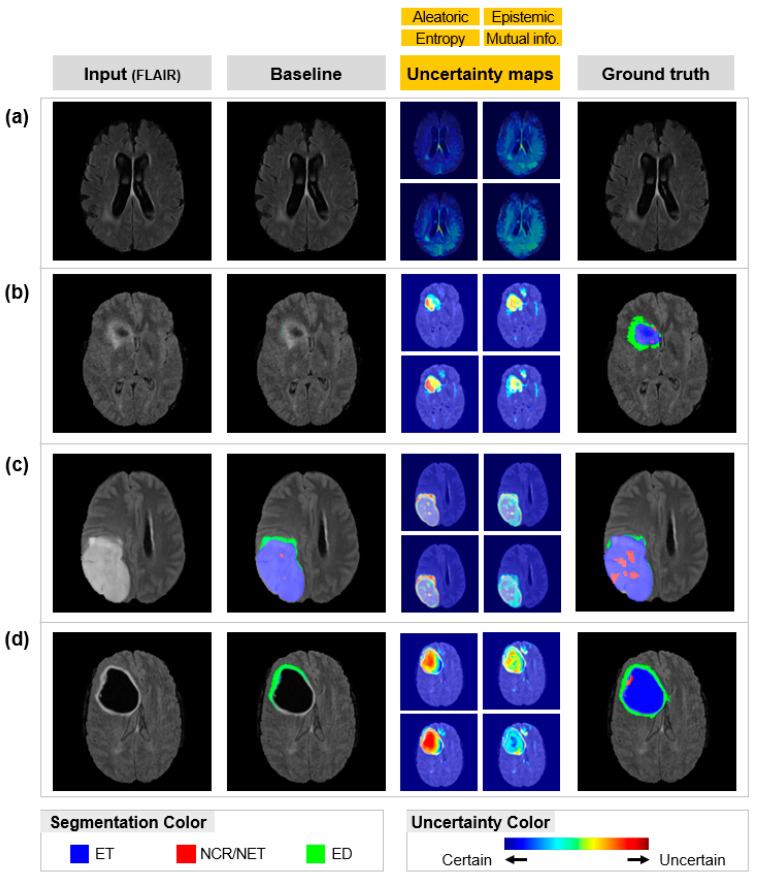
Uncertainty quantification results: Automated segmentation results by baseline model, corresponding uncertainty maps, image, and manual segmentation by experienced neurologists (ground truth). Each scan is from a different patient. The color code of the segmentation map and uncertainty map is described below. Red in the uncertainty map means an uncertain predictive area, and blue means a certain predictive area. ET = enhancing tumor; NCR/NET = necrotic and non-enhancing tumor; ED = peritumoral edema. (**a**) Totally correct prediction and UMs. (**b**) Totally incorrect prediction and UMs. (**c**) Slightly incorrect prediction and UMs. (**d**) Moderately incorrect prediction and UMs.

To find out the errors of the baseline model, we applied four different uncertainty measures, and the results are shown in Figure 4. It shows brain tumor segmentation and Uncertainty Maps (UMs), which show how uncertain the segmentations are. For example, red in the UMs denotes that those areas in the segmentation map are highly uncertain, and blue denotes that those areas are highly certain. These uncertainty measures, originated in statistics, were measured from data noise (Aleatoric and Entropy) and model imperfection (Epistemic and Mutual Information). These measures are widely utilized in various fields such as medical imaging to identify an algorithm’s reliability and to minimize its errors [14,20].

**Figure 5:**
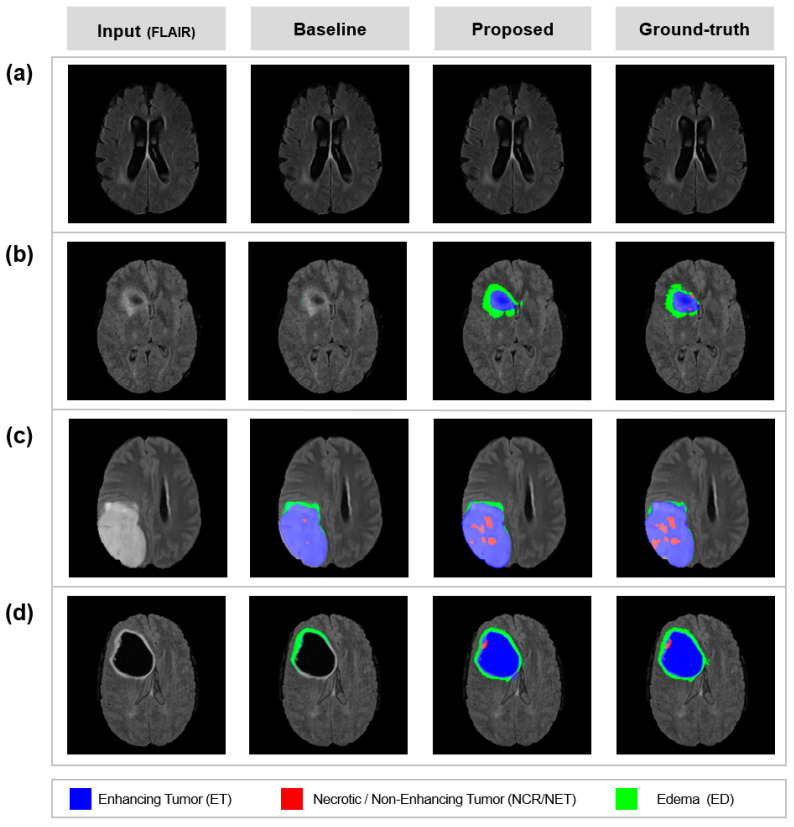
Uncertainty exploitation results: Automatic segmentation results by baseline/proposed method and manual segmentation by experienced neurologists (ground-truth). The images are subsequent results of Figure 4. The proposed method can exploit uncertainty maps to perform brain tumor segmentation. (**a**) scans with no tumor. (**b**) scans with ET and ED. (**c**) scans with all lesions. (**d**) scans with all lesions.

As a result, baseline segmentation maps, corresponding UMs, ground truth, and MRI images (FLAIR) are illustrated in Figure 4. Our UMs were corresponding maps that indicate the uncertainty of baseline segmentation results. The ground truth images are the manual segmentations performed by experienced neurologists. The color bar in Figure 4 represents the level of uncertainty. Red means that the baseline prediction is uncertain and blue means that the baseline prediction is certain. The UMs of Figure 4a have a blue color, indicating that baseline prediction was certain. On the contrary, the UMs of Figure 4b–d have various colors, including blue to red, indicating that some part of the baseline prediction was uncertain. Uncertain areas (Figure 5b) are more likely to be incorrect predictions than certain areas (Figure 5a). False negatives also tend to have highly uncertainty values (Figure 5d). Therefore, the UMs are significant indicators in showing the reliability of the prediction model. Moreover, tiny and confusable lesions, such as necrotic and non-enhancing tumors, show high uncertainty values (Figure 5c). Because Epistemic and Mutual Information measures model weakness, these maps can suggest what we can learn from the model.

### 3.2. Uncertainty Exploitation

In order to exploit UMs effectively, we designed a subnetwork module, UAM, which can be plugged into any kind of neural network. In this experiment, we plugged UAM into a well-known brain tumor segmentation model, MFNet and DMFNet, and compared the model’s performance with and without UAM. 

A qualitative comparison between the baseline and the proposed model is shown in Figure 5. The segmentation results of the model with UAM (proposed) corresponded well with ground truth. The model with UAM was effective in reducing significant errors, such as ET (Figure 5a,d). Moreover, tiny and confusable lesions such as NCR/NET were well predicted by using UAM (Figure 5c). The well-predicted lesions without UAM were maintained well even with UAM (Figure 5b).

The quantitative comparison between the baseline and the proposed model is summarized in Table 1. The proposed models achieved higher performances in ET (+3.15%) and TC (+0.58%) but had slightly lower performances in WT (−0.34%) when compared to the baseline models (DMFNet). In particular, the improvements in ET and TC were essential in precisely defining actual brain tumors that require resection in surgery. Even plugging an additional module, UAM, into the baseline models had almost no increase in inference time (FLOPs) or computing power (Params).

## 4. Discussion

In this work, we designed a general CNN framework to improve the performance of the model. Our proposed framework can (1) detect incorrect predictions by using uncertainty measures and (2) automatically correct wrong predictions with our proposed module, UAM. Our proposed framework improved the dice coefficient score of ET and TC by 3.15% and 0.58%, respectively. In addition, WT slightly decreased by 0.34% compared to the baseline model. Even if UAM was plugged into the baseline model, the computational costs and times were almost the same, increasing by 0.2% and 0.9%, respectively. Our framework can improve the model’s performance and achieve comparable results relative to the most accurate brain tumor segmentation models. This versatile framework can be applied to any form of CNN.

Uncertainty in outputs of the baseline model was estimated by four different well-known uncertainty measures [14,22,23,24]. UMs could effectively indicate false predictions. In particular, aleatoric and entropy measures were capable of capturing the model weakness caused by data imbalance. Epistemic and mutual information could represent model weakness caused by confusable and complex lesions. Uncertainty exploitation by our proposed module (UAM) was an effective method for modifying incorrect predictions. UAM can automatically correct the error instead of heuristic threshold filtering [14]. In order to reduce AI errors and improve its performance, uncertainty measures can be significant indicators.

Highly proliferating malignant cells need a larger blood supply and show reflect Gd-enhanced brain MRI than non-enhancing tumor lesions. Surgical resection and radiotherapy boundary in brain tumors usually include TC including ET and exclude peritumoral edemas [28]. Therefore, the exact demarcation of ET and TC is clinically important to assess treatment plans such as surgery and radiotherapy [28,29]. Because the proposed method achieved higher segmentation performance on ET and TC, as shown in Table 1, this method may contribute to the exact diagnosis of brain tumors.

We proposed a framework that can maximize the performance of any form of CNN by exploiting uncertainty measures. In this study, we adopted effective models, focusing on rapid diagnoses with lower costs [10] as baseline models. Although these models showed lower performance than the top-ranking model, we improved the performance of the models by our proposed framework. Moreover, the proposed model achieved comparable results relative to top-ranking models in the BraTS18 Challenge [11,12,13]. We maximized the baseline model’s ET and TC segmentation performance by applying our proposed framework, achieving dice coefficient scores of 83.27%, 85.12%, and 90.28% for ET, TC, and WT, respectively. This performance is a comparable result to the top three ranking models in the BraTS18 Challenge, in which the range of dice coefficient scores were 79.4~82.3%, 82.0~86.6%, and 90.0~91.0% for ET, TC, and WT, respectively [11,12,13]. Since our general framework can be applied to any form of CNN, it could be used strategically in various situations. Effective models can achieve comparable performance relative to high-performance models while maintaining effectiveness, such as real-time diagnosis. High-performance models can further maximize performance.

In conclusion, we designed a general framework to improve the accuracy of any form of CNN. The framework can detect incorrect predictions and correct them automatically. We demonstrated the effectiveness of our framework by using brain tumor segmentation. The segmentation performance of ET and CT was substantially improved by applying the proposed framework. 

## 5. Conclusions

In this work, we estimated the pixel-wise uncertainty of segmentation results. Moreover, we designed a new framework to exploit uncertainty information in order to upgrade the baseline segmentation model. The framework demonstrated the effectiveness of the public dataset, Multimodal Brain Tumor Segmentation Challenge 2018. In particular, this framework showed performance improvement in segmenting enhancing tumors that are typically small in size and difficult to segment, yet clinically important. We hope that our framework, highly optimized for the medical imaging domain, can be successfully applied to the medical field.

## Figures and Tables

**Table 1:** Evaluation results of baseline and the proposed method. DSC = dice coefficient; ET = enhancing tumor; TC = tumor core; WT = whole tumor; Params = number of model parameters; FLOPs = floating-point operations per second.

Model		MFNet	MFNet + UAM (Proposed)	DMFNet	DMFNet + UAM (Proposed)
DSC (%)	ET	79.91	82.56	80.12	83.27
	TC	84.61	84.93	84.54	85.12
	WT	90.43	89.56	90.62	90.28
Params (M)		3.19	3.20	3.88	3.89
FLOPs (G)		20.61	20.81	27.04	27.28

## Data Availability

The datasets analyzed during the study are available in the Multimodal Brain Tumor Segmentation Challenge 2018 (BraTS18) at https://www.med.upenn.edu/sbia/brats2018/data.html, accessed on 6 February 2022.
